# Management of traumatic subclavian artery injuries in a high-volume vascular surgery center in Iran

**DOI:** 10.34172/jcvtr.2020.24

**Published:** 2020-05-05

**Authors:** Niki Tadayon, Negin Yavari, Sina Zarrintan, Seyed Masoud Hosseini, Seyed Moahammad Reza Kalantar-Motamedi

**Affiliations:** ^1^Division of Vascular & Endovascular Surgery, Department of General & Vascular Surgery, Shohada-Tajrish Medical Center, Shahid Beheshti University of Medical Sciences, Tehran, Iran; ^2^Iranian Society of Vascular Surgery, Tehran, Iran; ^3^Research Department, Tehran Heart Center, Tehran University of Medical Sciences, Tehran, Iran; ^4^Cardiovascular Research Center, Tabriz University of Medical Sciences, Tabriz, Iran

**Keywords:** Subclavian Artery, Vascular Trauma, Proximal Control

## Abstract

***Introduction:*** Subclavian artery injury is an uncommon vascular trauma with potential morbidity and mortality. Management of subclavian artery trauma requires open and endovascular techniques and timely and efficacious decision is mandatory. We retrospectively reviewed traumatic subclavian artery injuries in a high-volume vascular surgery center in Iran.

***Methods:*** In a retrospective study, we assessed subclavian artery injuries during 6 years in ShohadaTajrish Medical Center. Background characteristics, type of incision, type of operation and outcome of patients were evaluated.

***Results:*** A total of 14 patients had subclavian artery injury (mean age 29.9 ± 13.4 years, 92.9% male). Trauma was in left and right sides in eight (57.1%) and six patients (42.9%) respectively. Arteriorrhaphy, interposition and ligation of injured artery was done in 7 (50.0%), 3 (21.4%) and 4 (28.6%) patients respectively. Associated nerve injury was present in six patients (42.9%). Endovascular proximal control was obtained in six patients (42.9%) prior to vascular exposure. Time of patient referral did not have significant association with shock or type of operation (*P* > 0.05).

***Conclusion:*** Although traumatic subclavian artery injuries are rare, its vascular exposures and reconstructions are of potential clinical concern. Endovascular interventions can facilitate proximal control. In addition, endovascular repair by covered stent is an alternative to open surgery.

## Introduction

Traumatic vascular injuries to subclavian artery is relatively rare with significant mortality and morbidity.^1^ The approach to these traumatized structures can be really challenging and requires effective management.^2^ Despite development of techniques and expansion of experiences it still remains one of the life-threatening injuries. Ultimate result of the treatment depends on the different approaches and concomitant injuries. Traditionally, penetrating or blunt subclavian artery injuries have been treated with open surgical repair. Direct surgical repair of subclavian artery injuries is rather difficult because of the surrounding structure and complex anatomy.^2^ Open surgery has been used in many years but it can still be associated with high rates of morbidity and mortality.^3^ Recent approach is endovascular technique with balloons for temporary control and covered stents for definitive repair.^2-4^ A limited number of cases have undergone endovascular repair and its safety and durability is still controversial. Herein, we reviewed outcomes of traumatic subclavian artery injuries at Shohada-Tajrish Medical Center. Our hospital is a high-volume trauma and vascular surgery center in Tehran, Iran.

## Materials and Methods

### Study population

From March 2013 to February 2019, a total of 14 patients (13 males and 1 female) who had subclavian artery injuries were admitted to Shohada-Tajrish Medical Center. Study variables consisted of demographic data, time of referral, type and mechanism of injury, operative management (completely open or hybrid), fasciotomy and types of incision. Distal pulses were absent in all 14 patients. CT angiography (CTA) was conducted in 8 (57.1%) of patients who were stable to reveal the site of injury. In addition, angiographic confirmation of subclavian artery injury was done in 6 patients (42.9%). All of the patients underwent open surgical repair for blunt or penetrating traumatic injuries to the subclavian arteries. In patients with hemodynamic instability, emergent surgical exploration was conducted to achieve immediate proximal and distal control of the site of exsanguination. Subsequent proximal control was done either by open surgical operation or endovascular balloon (length: 20-40 mm, diameter 8-10 mm) deployment. The size of the balloon was chosen based on the angiography. Informed consent was obtained from patients during their admission.

### Operative technique

**Figure 1:**
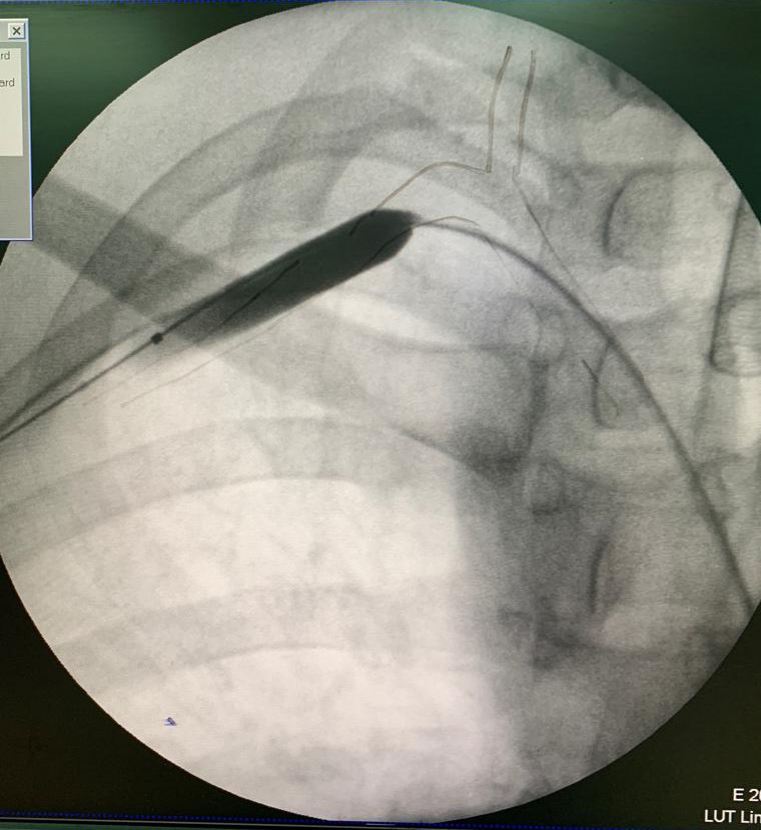
Proximal control of right subclavian artery injury with active extravasation on angiography.

**Figure 2:**
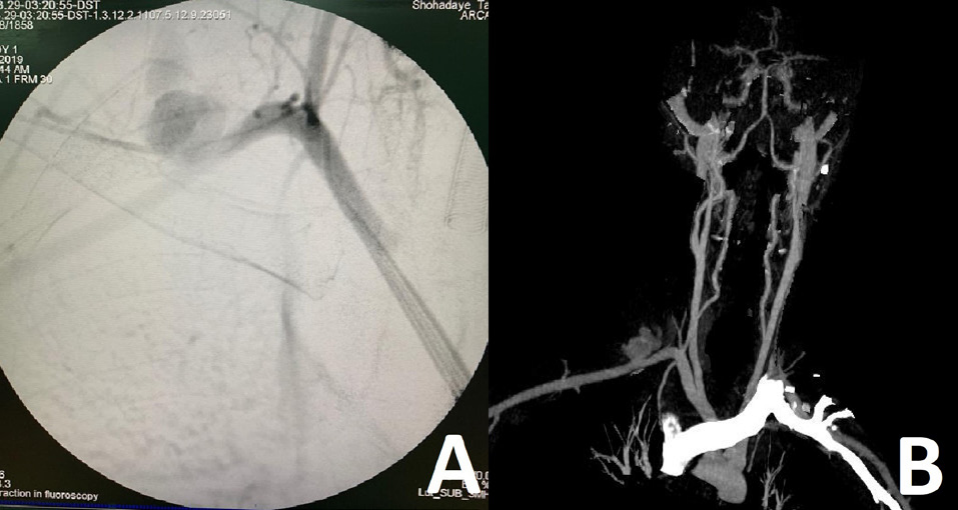
Right subclavian artery injury (A). Preoperative CT Angiography of injured right subclavian artery (B). Extravasation of the contrast material.

In a hybrid operating room and under general anesthesia, operations were conducted in supine position. Supraclavicular, infraclavicular, trapdoor or anterolateral thoracotomy incisions were used depending on the anatomical location of the injury. Arterial injuries were repaired by arteriorrhaphy, interposition by reverse saphenous vein graft or ligature and subsequent bypass. Proximal balloon control was conducted through access from common femoral artery or brachial artery via a 5F or 6F sheath. In femoral artery access, crossing to subclavian artery was conducted by Simmons and vertebral catheters with the guide of hydrophilic 0.035 guidewire. In brachial artery access, the contrast agent was administered from the sheath directly. Figure 1 shows arteriography revealed extravasation of subclavian artery injury. Six patients had the technique illustrated in Figure 2 to establish proximal balloon control from femoral or brachial accesses and wire access into injured part of the subclavian artery. Bleeding control was obtained through inflated balloon catheter by compressing the site of the injury to temporarily control the bleeding until surgical control and repair.

### Statistical analysis

Continuous variable was presented as mean with standard deviation (SD) and categorical variables were expressed as frequency and percentage. Statistical comparison between dichotomous variables was conducted by chi-square test. The comparison between continuous variables was conducted by *t* test. A *P* value less than 0.05 was considered to be statistically significant. All statistical analyses were conducted by SPSS 24.0.

## Results

A total of 14 patients (mean age 29.9 ± 13.4 years, 92.9% male) had subclavian artery injury during 6 years. Baseline characteristics and associated injuries are shown in Table 1. Eight number of the injuries (57.1%) were in left side and six of them (42.9%), were in right side. The mechanism of injury was blunt trauma in four patients (28.6%) and penetrating trauma in ten patients (71.4%). There was an associated vein and nerve injuries in four (28.6%) and six (42.9%) patients, respectively. All the patients were successfully managed and discharged.

**Table 1:** Background variables and associated injuries of patients with traumatic subclavian injury

**Variable**	
Age, mean (SD)	29.93 (13.44)
Gender, male, No. (%)	29.93 (13.44)
Time of referral, No. (%)	
<6 hours	8 (57.1%)
6-12 hours	3 (21.4%)
12-24 hours	2 (14.3%)
>24 hours	1 (7.1%)
Trauma type, No. (%)	
Blunt	4 (28.6 %)
Stab	10 (71.4%)
Gunshot	0 (0)
Shock class III or IV, No. (%)	6 (42.9%)
Vein injuries, No. (%)	
Yes (ligated)	4 (28.6 %)
No	10 (71.4%)
Nerve injury, No. (%)	
Yes	6 (42.9%)
No	8 (57.1%)
Associated injury, No. (%)	
Spinal	2 (14.3)
Limb	1 (7.1)
No	11 (78.6)
Fracture, No. (%)	
Yes	3 (21.4)
No	11 (78.6)

Supraclavicular and infraclavicular incisions were done to repair subclavian artery injuries in eleven (78.6%) and one (7.1%) patients respectively. There was also one case of thoracotomy (7.1%) and one case of trapdoor incision (7.1%) based on physical examination and preoperative CTA results. Arteriorrhaphy and ligation of injured arteries were done in 7 (50.0%) and 4 (28.6%) patients respectively. Endovascular proximal control was obtained in six patients (42.9%) prior to vascular exposure. Time of patient referral did not have significant association with shock or type of operation (*P* > 0.05).

Mean hospital admission days was 8.5 ± 5.0 days. Mean intensive care unit (ICU) admission days was 1.2 ± 1.3 days. Ward admission and ICU admission days were significantly more in patients with blunt trauma than in the patients with penetrating trauma (*P* values were 0.025 and 0.001 respectively). However, the associations between time of referral of patients and total and ICU admission days were not statistically significant (*P* > 0.05; Table 2).

**Table 2:** Operative details of patients with traumatic subclavian injury

**Variable**	
Operation type	
Arteriorrhaphy	7 (50.0%)
Interposition	3 (21.4%)
Ligature & Bypass*	4 (28.6%)
Incision	
Supraclavicular	11 (78.6%)
Infraclavicular	1 (7.1%)
Thoracotomy	1 (7.1%)
Trapdoor	1 (7.1%)
Endovascular proximal control	
Yes	6 (42.9%)
No	8 (57.1%)
Fasciotomy of upper limb	
Yes	1 (7.1%)
No	13 (92.9%)
Admission days	8.5 ± 5.0
Intensive care unit length of stay	1.2 ± 1.3

*Bypass was conducted in second operation in the same admission.

## Discussion

In the present study, our patients experienced penetrating or blunt subclavian artery injuries and regarding the potential significance of arterial injuries, prompt and precise decisions for diagnosis and management of injuries were considered.

Recent studies demonstrated that endovascular stent-graft repair is used in both elective and emergent cases of traumatic subclavian artery injuries.^5-7^ Open surgery is still a method of choice and definitive modality to treat subclavian artery injuries^8,9^ but progress in endovascular techniques and increased experience in endovascular therapy lead to increased use of covered stents.

Injured subclavian artery is difficult to expose due to hematoma formation and anatomical complexity. Thus, approaching to the injured site by guidewire and deployment of stent-graft is a simpler method. However, one of the potential disadvantages of endovascular approach in penetrating trauma is subclavian artery transection due to difficultly in crossing the guide wire either by femoral or brachial access, especially when it is associated with huge hematoma.^4,8-11^ While surgery in blunt subclavian artery injury is a difficult procedure, endovascular approach decrease the injuries to the adjacent structures.^1,5,8^

Hemodynamically unstable patients should undergo immediate operative intervention, to determine the location, severity of the injury and to repair subclavian artery injury.^4^ Because endovascular approach requires significant time to perform angiography and conduct endovascular repair, its use in patients with hemodynamic compromise is limited to proximal control by balloon.^4^ Despite the fact that endovascular repair in unstable patients is almost contraindicated but it is more favorable in such cases due to have shorter operative duration and lower blood loss^6,9,10,12,13^ and it also avoids thoracotomy which is desirable in a number of cases.^1,14^

Difficult surgical exposure of subclavian artery can be challenging and sometimes the lesion cannot be controlled by compression especially when the injury is in a retro-clavicular position. In these cases, balloon tamponade technique is used to temporarily control the bleeding until transferring the patient to the operating room.^5^ In our cases, tamponade compression in emergency department was not necessary but endovascular balloon was used in six patients at the proximal subclavian artery to achieve a rapid control of bleeding

Anatomic differences in right and left subclavian artery requires different approaches. Left sided subclavian artery injury can be exposed through supraclavicular, anterolateral thoracotomy and trapdoor incision and because it has its own origin from aorta; therefore, concern about ipsilateral carotid artery embolism or occlusive events are lower and endovascular techniques are used more safely to repair left subclavian injuries.^4^ On the other hand, right subclavian artery needs to be entirely open via extra-thoracic approach or median sternotomy.^4,15^ Due to extensive incisions to expose the injured area entirely, endovascular approach might offer less invasive approach to this artery but possible limitations and controversies should be considered.^8^ Surgery can be performed through median sternotomy, supraclavicular, infraclavicular or via thoracotomy and trapdoor approach depending on the location of subclavian artery injury. Controlling the proximal injury can be achieved by supraclavicular incision or median sternotomy based on the location of the injury. Previous studies demonstrated that the combination of median sternotomy and clavicular incision can provide better exposure for both sides^16^ to avoid trapdoor incision because it is accompanied by severe pain, excessive bleeding and respiratory complications.^4^ Only one patient in our study had thoracotomy and one patient had trapdoor incision. In some studies, endovascular approach in selected patients with shock were also amenable but a hybrid operation room should be considered to switch to open surgery if necessary.^4^

Endovascular approach may be beneficial in some candidates including stable patients or unstable ones with expert vascular surgeons; in presence of hybrid operation room, and distinct focal injuries. There are several complications with endovascular approach and also few relative contraindications.^12,17^ Contraindications are inadequate distal or proximal landing zones, sustained hypotension, unresponsive to fluids or blood resuscitation, artery transection or occlusion and compartment syndrome.^4,12,17^ Injuries that are not accessible easily or when an experienced trauma or vascular surgeon is not available, endovascular therapy can be used by guide wires and covered stents to repair the injuries.^17,18^ Conversion to standard open surgery may be necessary when complications occurs such as hematoma or pseudoaneurysm. If the guidewire cannot reach to the injured area whether via femoral or brachial route, open surgery will take place. Re-operation may happen due to stent graft fracture or dislocation because of repeated of movement in this area and extrinsic compression.^13,17,19^ Another complication of endovascular technique is stent thrombosis post operatively due to increased hypercoagulability state or frequent contraindication in using anti-thrombosis medication, embolization due to transection of the artery and neointimal hyperplasia.^9,12,13^ Distal embolization in vertebral artery origin or in right side subclavian artery bifurcation due to provocation of thrombosis into common carotid artery and subsequent cerebral infarction has been a hazardous complication .^13^ Hyperplasia can also raise concern about patency of stent in short and long-term follow-ups.^18^ It is possible that polytetrafluoroethylene covered stent can reduce the risk of neointimal hyperplasia but further clarification is still necessary.^19^

## Conclusion

Despite the fact that subclavian artery injury happens infrequently, its injuries and reconstructions are one of the potential clinical and surgical concerns. A number of reports suggest that endovascular repair is one of the possible methods to restore the arterial flow in subclavian artery but indications and management guidelines are still lacking and thus, open repair is still a method of choice. It is also possible to conduct hybrid therapies. Preoperative proximal control by balloon deployment diminished preoperative and intraoperative bleeding and improves outcome. Preoperative planning regarding the most suitable incision and also possibility of endovascular intervention can be achieved by CTA in stable patients.

## Competing interests

None declared.

## Ethical approval

The protocol of this study was approved by Shohada-Tajrish Medical Center, Shahid Beheshti University of Medical Sciences, Tehran, Iran. It was a single-institutional retrospective study and departmental and institutional approvals were obtained for publication.

## Acknowledgments

The authors acknowledge all general surgery residents who were involved in case of the presented patients. The authors also thank Dr. Annis Shahnaee for her useful comments during the preparation of this article.
